# First Semi-Annual Report of the Jacksonville Surgical Infirmary

**Published:** 1868-10

**Authors:** David Prince, Wm. H. H. King, M. A. Bosworth, Agnes W. Hill

**Affiliations:** Proprietor and Attending Surgeon; Resident Surgeon; Matron; Chief of Subsistence


ARTICLE XLIII.

### FIRST SEMI-ANNUAL REPORT OF THE JACKSONVILLE SURGICAL INFIRMARY.

OFFICERS IN IMMEDIATE CHARGE OF THE INSTITUTION.

DAVID PRINCE, M.D., Proprietor and Attending Surgeon.
WM. H. H. KING, M.D., Resident Surgeon.
MISS M. A. BOSWORTH, Matron.
MRS. AGNES W. HILL, Chief of Subsistence.

In accordance with an announcement issued last fall, this enterprise went into operation about the beginning of the present year, and while its success has been encouraging to its managers, it is believed to have been satisfactory to all who have sought the benefits which it was designed to bestow.

It is easily understood that those who go away from home for medical treatment and operations, receive the benefit which they seek at a disadvantage, if forced to board around wherever a vacancy may be found. They can only be seen by their medical advisers by special visits, while in an institution, a more frequent examination of the case is practicable and all the appliances to be used in the treatment are at hand.

Besides, it is practicable to keep on hand and to employ various apparatus which it would be inconvenient to set up in private houses and to move about from place to place.

It is also practicable to secure more skilled and efficient nursing, in an institution conducted for the purpose of caring for the sick and the disabled, than is possible under other circumstances.

Another feature which secures special advantages for the winter season, is the warming of the house by furnaces in the basement, so that a pleasant and equable temperature is secured at the same time that a considerable degree of ventilation, always desirable, is rendered unavoidable. The colder the weather is, the greater the amount of heated air introduced from without through the furnace chambers. This of course displaces so much air already in the room, which escapes by the openings, whether windows, doors and cracks, or openings specially provided. An arrangement is also made, by which all impurities can be taken out of the incoming atmosphere, so that the air introduced into the rooms through the furnace is purer than the outside atmosphere. On the contrary, where stoves are employed, the colder the weather is the worse the ventilation on account of the temptation to stop all the cracks through which the cold air strives to enter the room.

There is, at the same time, a temptation to build as few fires as possible, so that in going from one room to another, there is the danger and discomfort of passing through cold or variously heated atmospheres. The building is thus specially adapted for the winter residence of feeble persons and those afflicted with pulmonary consumption.

Under the direction of Miss Bosworth, the Matron, the greatest possible approach to home comfort is secured, and it is a pleasant recollection, that several children that have been left here by their parents have said on leaving, that they had enjoyed themselves better than if they had been at home.

The department of subsistence is presided over by Mrs. Hill, a lady of great experience in the art. While no attempt is made to imitate the variety of hotel cooking, especial pains is taken to render plain food palatable and digestible.

A brief notice of some of the cases which have been treated in the institution may be of interest to medical men.

Flexed Knee-Joint.—Of this mal-position, six cases have been under treatment with entire success in bringing the limb to a straight position.

One of the cases was in the acute stage of synovitis occurring spontaneously, or without a wound. Sleep was greatly disturbed by spasmodic contraction of the muscles, and the mal-position was rapidly increasing. The limb was gradually restored to the straight position by a weight acting night and day over a pulley. As the joint surfaces ceased to press upon each other, the spasmodic muscular contractions disappeared. The ability to be moved was greatly aided by a splint, flanked with pasteboard, and worn behind. After six months' treatment, the discharge from the joint continues, but the limb is straight, and the joint movable and free from pain while the weight is on, but if the weight is left off for some time, pain in the knee returns. The probability is that recovery will be effected without deformity or lameness, but time is necessary for the gradual subsidence of inflammation and suppuration.

The remaining five cases were the results of inflammation in which the soreness had abated, or altogether subsided. In all these cases force was first applied under ether, and afterward gradual extension by means of a weight varying from five to fifteen pounds. In two of the five cases, the flexors of the leg were divided by a bistoury, but it is doubted whether the final result was any more quickly attained by this means. In one of the cases the wounds made in the process became the seat of a troublesome spreading erysipelas, with burrowing of pus among the muscles. All this would probably not have occurred, if the skin had not been wounded. The final result, however, was very satisfactory.

As the resistance after the subsidence of inflammation is usually in the short ligaments and adhesive attachments more than in muscular force, the division of the muscles removes only a minor difficulty.

In cases of extreme flexion, it is important that the gradual extension should first be in such direction as to separate the joint surfaces, waiting for a later period of the treatment to straighten the limb, otherwise, portions of the joint surfaces are pressed together with great violence by the lever of the leg, and the pain of this pressure becomes insupportable. One case of stiff knee in the straight position, resulting from destructive inflammation following a severe injury, seemed to be much benefited by treatment, but with the discontinuance of extension and passive motion, the parts resumed their tightness and immobility. In the first application of force in this case, the skin was ruptured transversely over the ligament of the patella; but by means of silver sutures, a dressing of permanganate of potash and the straight position for a few days, union by adhesion was secured. When the soft tissue of a joint is to a considerable extent destroyed, there is very little probability of success in restoring its function.

Two interesting cases of cheiloplasty have been under treatment, one of which is the completion of the case on page 179 of the Transactions of the Illinois State Medical Society for 1867, and page 67 of the book as published by Lindsay & Blakiston. One of the important points in plastic operations, is to have a good arterial supply to the parts which it is desired should unite.

Chronic ophthalmia with persistent congestion, vascular cornea and granular lids and subsequent inversion of the ciliary margins, has presented several interesting cases. The division of the ciliary bands as practised by Dr. Hildreth, of Chicago, and the subsequent depression of the pulse for several days by means of veratrum viride have seemed to afford a starting-point from which to commence a restoration of the normal vascular condition. Some aqueous humor is usually lost at the time of the operation, which also aids in the relief of pressure, and though the liquid is soon replenished, a temporary relief has interrupted the succession and made a new action possible. Citrine ointment made with cod-liver oil, as recommended by Dr. Williams, of Cincinnati, has been found very valuable for granular lids, especially when following the treatment just indicated.

For inversion of the ciliary margins of the lids, with shortening of the palpebral fissure, the method of implanting behind the outer portion of the upper lid a portion of integument, has not lost its favor. A full description of this method may be found in the work on Plastic Surgery already referred to. The dextrous performance of this operation leaves no deformity whatever. It may be practised at the same time that the ciliary bands are divided. The removal of a strip of integument just above the ciliæ, after the method of Des Marres, is rendered effective by the operation for the elongation of the palpebral fissure.

Two operations for artificial pupil have been performed during the half year with perfect result. The new method of approach through the margin of the sclerotic has been followed.

In one case a gold tube has been inserted for closure of the nasal duct. This method, in the writer's experience, has succeeded in every instance in which the lachrymal sac was enlarged by accumulation of pus; but where this was not the cause, it has uniformly failed, probably because the sac closed down over the top of the tube and obstructed it.

Cases of enlarged or sub-involuted uterus, mal-positions and ulcerations of this organ, have also been under treatment in the Infirmary.

One case of radical operation for hernia according to Chisholm's method has proved completely successful. The amount of peritoneal inflammation was alarming for a few days, so as to raise the question whether the operation is justifiable.

An interesting case of lithotomy by the median method, in which three very large calculi were removed without incision of the prostate, but with laceration of the perineum into the rectum, demonstrates that incision of the prostate for the removal of a stone from the bladder is unnecessary.

The case also shows that a more extensive division of the perineum should be practised than is contemplated by Allarton's method where the stone is large. A recto-urethral fistula resulted from this operation on account of which several plastic operations have been performed, but thus far without complete success.

A case of compound comminuted fracture of the femur has demonstrated the great comfort secured by suspending the limb, permitting the patient to move his body at will without disturbing the relations of the fragments. A simplification of Dr. Hodgen's suspension splint has been employed, the extension being effected by means of a strip of adhesive plaster on either side of the leg, attached to the foot-piece, and the counter-extension being the weight of the body pulling down upon the limb which is upon the inclined plane of the suspended apparatus. Lint soaked in solution of permanganate of potash prevented the suppuration which otherwise would have commenced at the surface through the putrefaction of the fluids, thence spread ing decomposition through the effusion in the deep parts of the wound. The shortening immediately after the injury was four inches. The patient recovered with a final shortening of three-quarters of an inch. A good result under the circumstances.

From comparative trials made with permanganate of potash and carbolic acid in erysipelas, putrefaction and complications of wounds, permanganate is believed to be greatly superior to carbolic acid and to everything else which has been tried. It is applied over wounds and upon erysipelatous surfaces in nearly a saturated solution. Over wounds it is best applied by laying on a portion of lint saturated with the solution. This is probably the best application to make to bullet wounds and those attendant upon compound fractures. By preventing the putrefactive change in the exudations and effusions, the extension into them of the organizing process is greatly favored. Care should be taken not to apply the solid salt, as it acts as a caustic.

The only death which has occurred in the Infirmary during the first half-year of its existence was that of an old man with enlarged and suppurating prostate. Death occurred at the end of ten days from his reception and twenty days from the inflammatory attack.

Great difficulty had been experienced in introducing the catheter, which was done three times a day, and the P. M. revealed the existence of false passages which had been supposed to be among the causes of the difficulty of catheterism.

The convenience and the success with which cases have been managed in this institution, are an assurance of the propriety and the necessity of the undertaking.

*July, 1868.*

